# Case Report: A case of thoracic schwannoma: diagnostic work-up of an incidentally detected mediastinal lesion

**DOI:** 10.3389/fmed.2026.1891368

**Published:** 2026-09-14

**Authors:** Domenico Cristiano, Anna Annunziata, Antonietta Coppola, Marcellino Cicalese, Pasquale Imitazione, Maurizia Lanza, Antonella Marotta, Giuseppe Fiorentino

**Affiliations:** UOC Respiratory Physiopathology and Rehabilitation, AORN dei COLLI, Naples, Italy

**Keywords:** benign lesion, EBUS-TBNA, mediastinal schwannoma, PET/CT, thoracic imaging

## Abstract

Schwannomas represent the most common neurogenic tumors of the posterior mediastinum. Although typically slow-growing and asymptomatic, degenerative changes such as cystic transformation may alter their imaging appearance and mimic necrotic lymphadenopathy or malignant mediastinal masses. A 61-year-old man underwent chest CT for respiratory symptoms. Careful image review revealed an incidental subcarinal mediastinal lesion initially suspected to represent necrotic lymphadenopathy. Further evaluation with contrast-enhanced CT, 18F-FDG PET/CT and endobronchial ultrasound-guided transbronchial needle aspiration (EBUS-TBNA) was performed. PET imaging demonstrated moderate FDG uptake while EBUS-TBNA cytology was non-diagnostic. Progressive enlargement of the lesion and the onset of compressive symptoms led to surgical resection. Histopathological examination confirmed a benign mediastinal schwannoma with cystic degenerative changes. This case highlights the diagnostic pitfalls associated with mediastinal schwannomas and underscores the complementary role of multimodal imaging and multidisciplinary assessment in the diagnostic work-up of a mediastinal lesion.

## Introduction

Neurogenic tumors account for approximately 20–30% of mediastinal neoplasms in adults and are the most common tumors arising in the posterior mediastinum (1). Among these, schwannomas are the most frequent subtype and originate from Schwann cells of peripheral nerve sheaths.

Mediastinal schwannomas are typically benign, encapsulated tumors characterized by slow growth. They most commonly arise from intercostal nerves, the sympathetic chain or spinal nerve roots and are often discovered incidentally during imaging examinations performed for unrelated clinical conditions (2).

On computed tomography (CT), schwannomas usually appear as well-circumscribed masses with soft-tissue attenuation and mild to moderate contrast enhancement. However, secondary degenerative changes such as cystic degeneration, hemorrhage or calcification may alter their radiological appearance and simulate necrotic lymphadenopathy or malignant mediastinal tumors (3).

Although contrast-enhanced CT remains the primary imaging modality for evaluating mediastinal masses, complementary techniques such as positron emission tomography (PET/CT) and endobronchial ultrasound (EBUS-TBNA) can provide additional metabolic and cytological information. Nevertheless, definitive diagnosis often requires histopathological confirmation following surgical resection.

We report a case of an incidentally detected posterior mediastinal schwannoma initially interpreted as necrotic lymphadenopathy, illustrating the role of multimodal imaging in the diagnostic work-up.

### Case presentation

A 61-year-old male former heavy smoker (70 pack-years) presented to our respiratory clinic with cough, worsening dyspnea and symptoms suggestive of gastroesophageal reflux disease.

At clinical evaluation, the patient presented with a chest CT scan performed a few weeks earlier. Careful re-evaluation of the images, particularly using mediastinal window settings, revealed an incidental lesion in the subcarinal region that had not been previously reported and was unknown to the patient (Figure 1).

**Figure 1:**
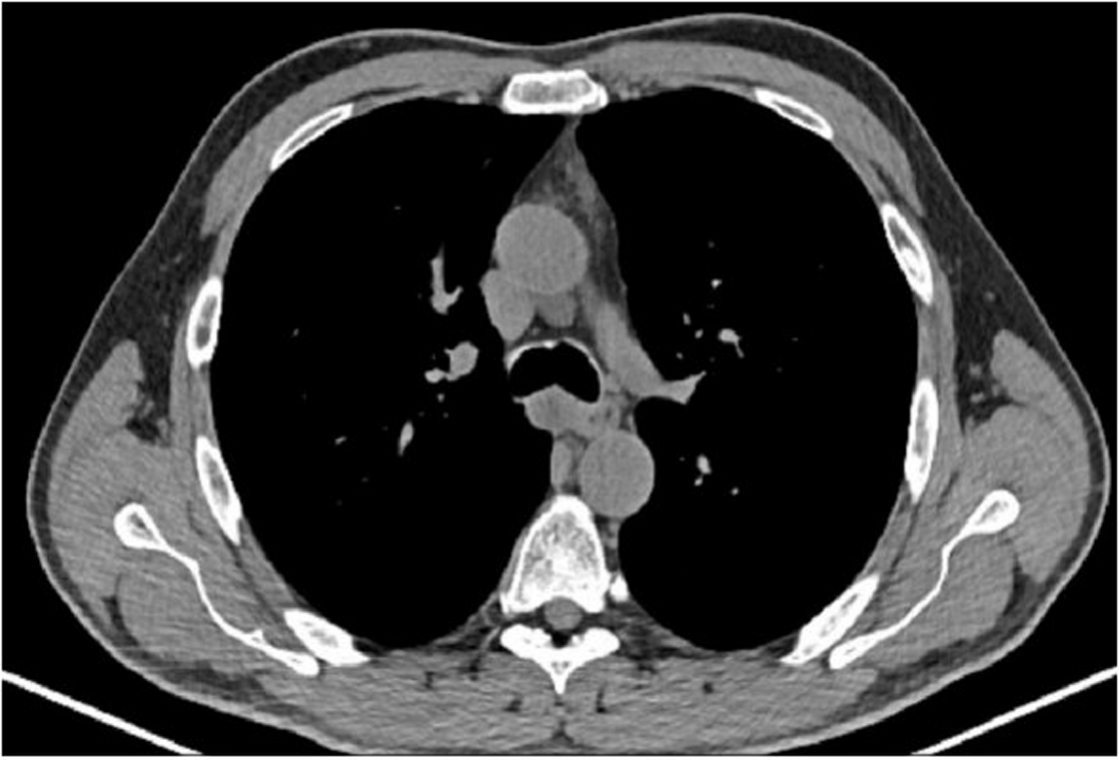
Chest CT scan showing a subcarinal lesion.

Coronary CT angiography performed for evaluation of chest pain confirmed the presence of a well-defined subcarinal mass measuring approximately 22 × 19 mm with central hypoattenuation suggestive of necrotic or cystic content and partial compression of the esophageal lumen (Figure 2).

**Figure 2:**
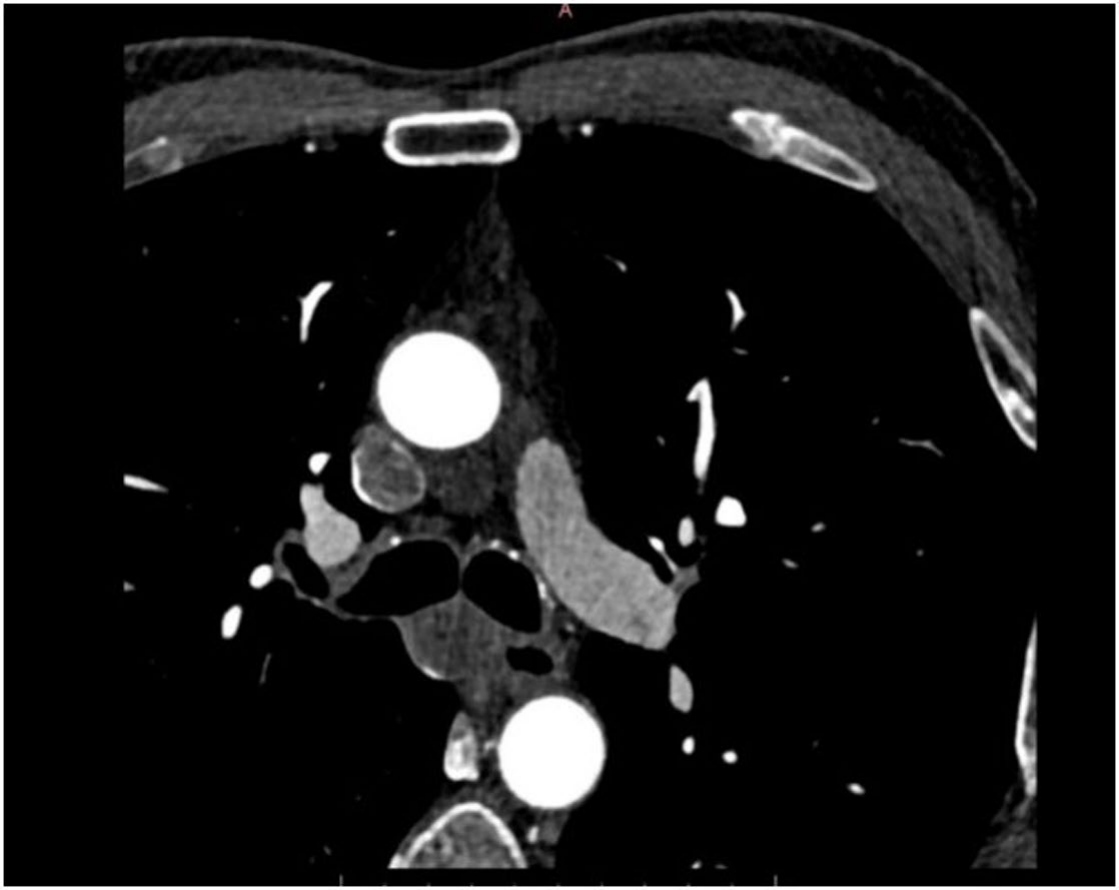
Coronary CT angiography demonstrating a subcarinal mass measuring approximately 22 19 mm.

To further evaluate the lesion, whole-body 18F-FDG PET was performed. The examination demonstrated moderate metabolic activity within the mediastinal lesion (SUVmax 4.81) (Figure 3).

**Figure 3:**
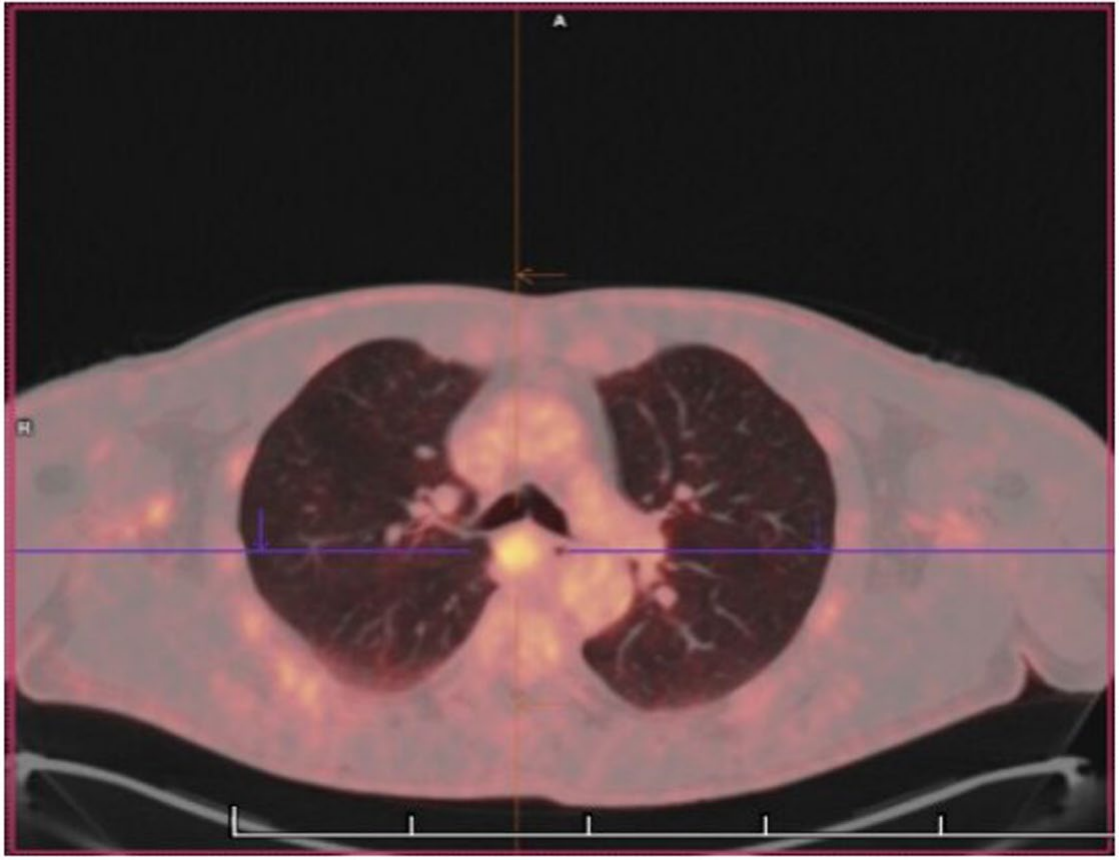
18F-FDG PET/CT scan showing metabolic activity in the subcarinal lesion.

The case was discussed in a multidisciplinary team involving radiologists, nuclear medicine physicians and pulmonologists. Given the PET-positive mediastinal lesion and the absence of clinical or laboratory findings suggestive of malignancy, endobronchial ultrasound-guided transbronchial needle aspiration (EBUS-TBNA) was performed.

Endosonographic examination revealed a well-circumscribed round lesion with anechoic content and marked posterior acoustic enhancement, without internal septations or solid components (Figure 4).

**Figure 4:**
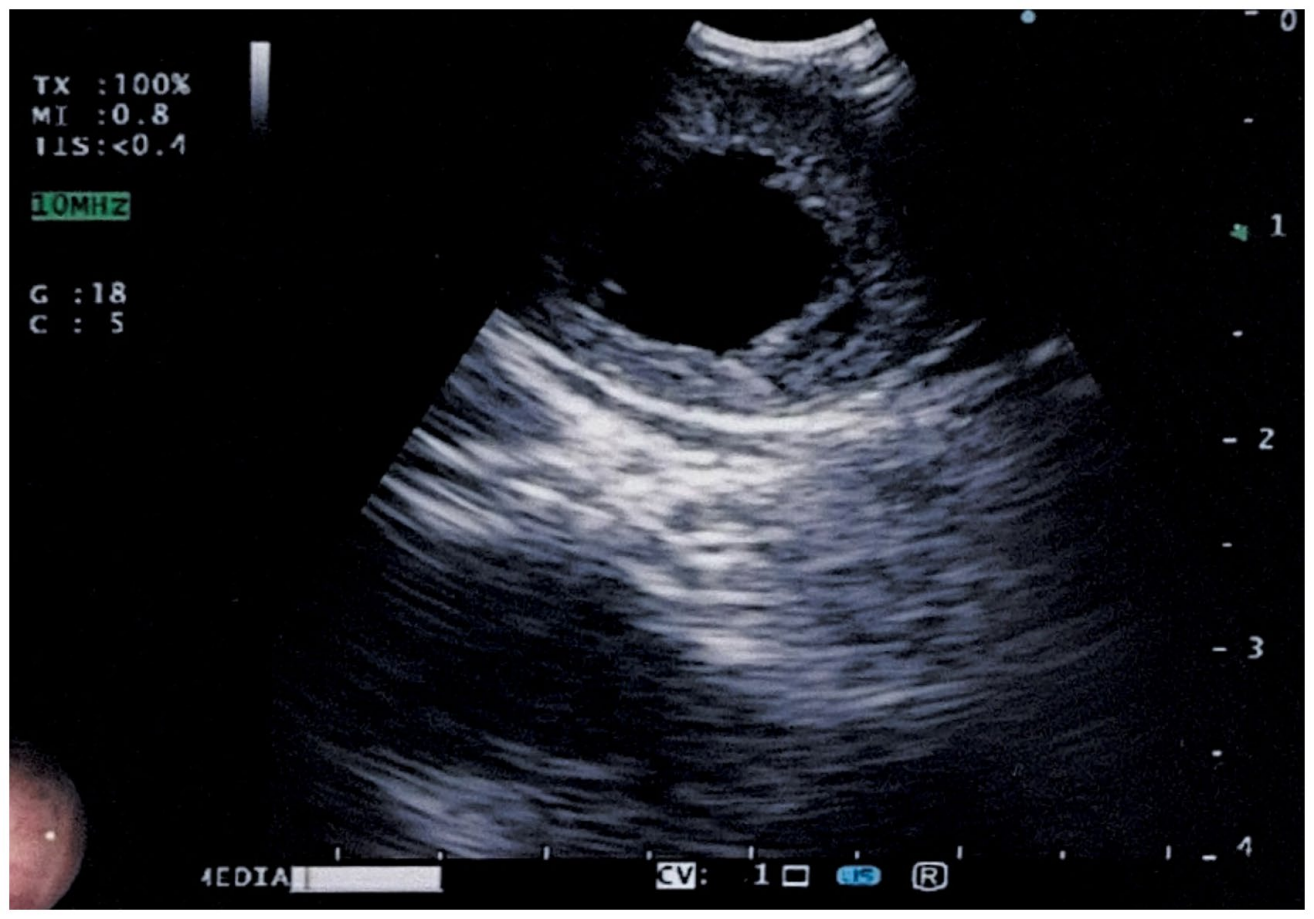
Endosonographic examination showing a round lesion.

Cytological analysis excluded malignant cells but was non-diagnostic, revealing blood material, lymphocytes and fibrous fragments (see Table 1).

**Table 1:** Chronological timeline of diagnostic work-up and treatment.

Time point	Clinical event
*January 2024* Initial presentation	Incidental subcarinal mediastinal lesion identified during chest CT image review of a 61-year-old former heavy smoker
*March 2024* Coronary CT angiography	Subcarinal lesion with central hypoattenuation and partial esophageal compression
*June 2024* First 18F-FDG PET/CT	Moderate FDG uptake (SUVmax 4.81)
*June 2024* Multidisciplinary discussion	Radiological and clinical assessment by pulmonologists, radiologists and nuclear medicine specialists
*September 2024* EBUS-TBNA	Cystic lesion identified; cytology non-diagnostic and negative for malignancy
*March 2025* Follow-up visit	Chest CT showed enlargement to 30 × 22 mm with increased cystic component.
*April 2025* Pre-operative PET/CT	Decreased metabolic activity (SUVmax 3.22)
*July 2025* Robotic-assisted thoracoscopic surgery	Complete resection of posterior mediastinal mass. Histopathological examination showed benign schwannoma with Antoni A/B areas and extensive cystic degeneration
*December 2025* Follow-up visit	Clinical improvement and absence of surgical complications

Because of inconclusive findings, radiological follow-up was adopted.

Follow-up contrast-enhanced CT demonstrated progressive enlargement of the lesion to approximately 30 × 22 mm with an increased central hypodense component (Figure 5). The patient also reported worsening gastroesophageal reflux symptoms likely related to esophageal compression.

**Figure 5:**
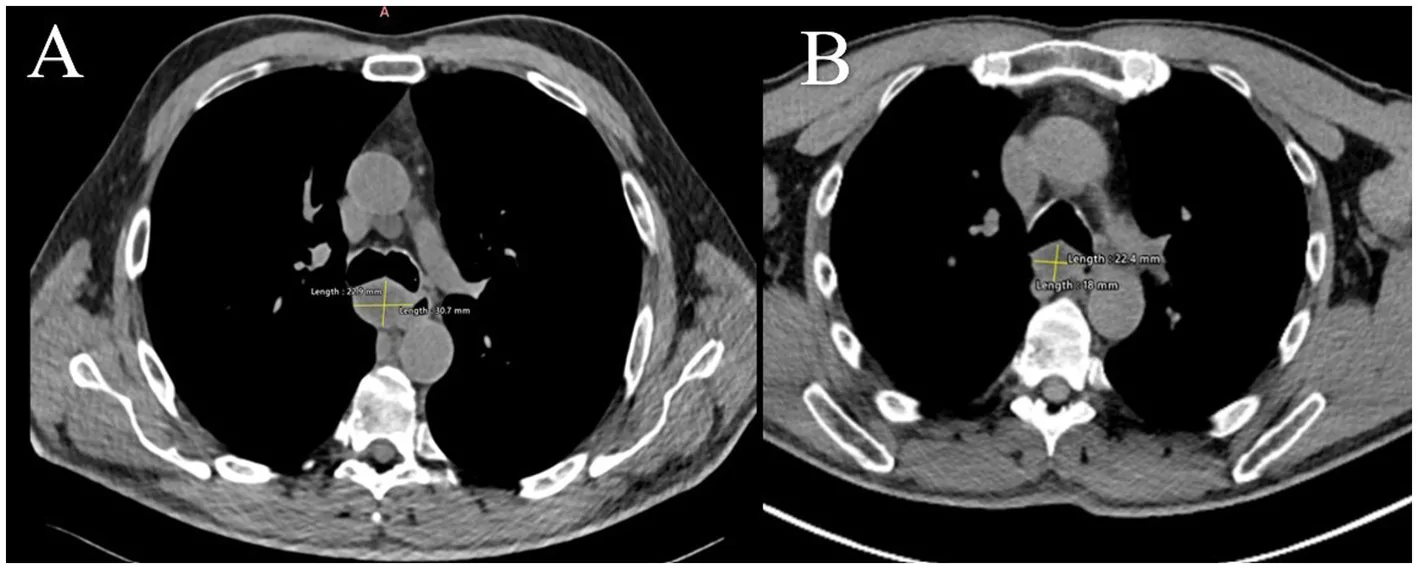
Comparison between follow-up chest CT **(A)** and previous CT images **(B)**, demonstrating enlargement of the lesion.

Given the radiological progression and symptom evolution, surgical resection was recommended.

A repeat PET/CT prior to surgery showed reduced metabolic activity of the lesion (SUVmax 3.22), suggesting low biological aggressiveness.

The patient underwent robot assisted (Da Vinci Robot) right thoracoscopic surgery using a triportal approach. Complete resection of the posterior mediastinal mass was achieved without complications.

Macroscopic examination revealed a partially cystic mass measuring approximately 4 × 3 × 2 cm containing reddish mucoid material. Histopathological analysis demonstrated a well-encapsulated tumor composed of spindle cells arranged in fascicles with alternating hypercellular (Antoni A) and hypocellular (Antoni B) areas (Figure 6). Extensive cystic degenerative changes were present.

**Figure 6:**
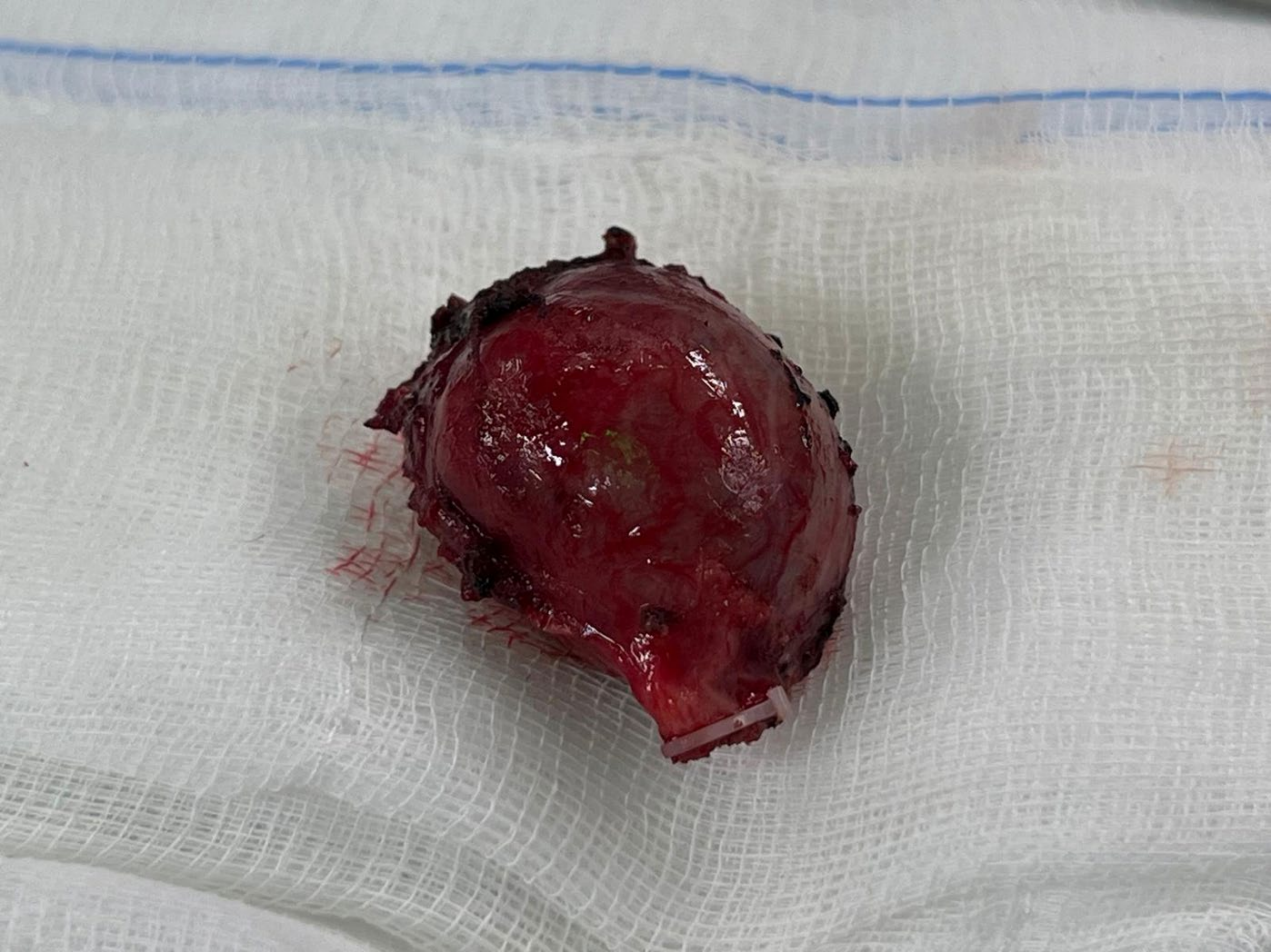
cystic mass removed.

These findings confirmed the diagnosis of mediastinal schwannoma.

## Discussion

Schwannomas are benign peripheral nerve sheath tumors characterized by slow growth and encapsulation. In the thorax, they most commonly arise from intercostal nerves, spinal nerve roots, or the sympathetic chain and account for the majority of neurogenic tumors of the posterior mediastinum. Although usually asymptomatic, larger lesions may cause symptoms due to compression of adjacent structures (4).

Contrast-enhanced CT remains the cornerstone imaging modality for mediastinal mass evaluation. Typical CT findings include a well-defined soft-tissue mass with moderate enhancement. Typical schwannomas appear as well-circumscribed lesions with homogeneous or heterogeneous enhancement.

The present case illustrates the diagnostic challenges associated with mediastinal schwannomas presenting with cystic degeneration. Degenerative changes including cyst formation, hemorrhage, calcification, and myxoid degeneration are well-recognized features of long-standing schwannomas and may substantially alter their radiological appearance (3). In our patient, the presence of a central hypodense component initially raised suspicion for necrotic lymphadenopathy, highlighting the potential for misdiagnosis.

Endobronchial ultrasound-guided transbronchial needle aspiration (EBUS-TBNA) has become a widely used minimally invasive technique for evaluating mediastinal lymph nodes. Nevertheless, its diagnostic yield may be limited in cystic lesions or benign tumors, as cytological sampling may not provide adequate tissue architecture for definitive diagnosis (5).

PET/CT provides functional information based on glucose metabolism and plays an important role in oncologic imaging. However, FDG uptake is not specific for malignancy. Several benign lesions, including schwannomas, inflammatory processes and granulomatous diseases, may demonstrate moderate FDG uptake (6–8).

In the present case, the combined findings of CT, PET/CT and EBUS-TBNA were not sufficient to establish a definitive diagnosis. Surgical resection therefore served both diagnostic and therapeutic purposes.

An additional aspect of this case was the progressive onset of gastroesophageal reflux symptoms. Esophageal compression has been described in isolated reports of esophageal and paraesophageal schwannomas and may manifest with atypical upper gastrointestinal symptoms, including reflux, dysphagia, or retrosternal discomfort (9, 10). The temporal association between lesion enlargement and symptom worsening in our patient supports a compressive mechanism.

Complete surgical excision remains the treatment of choice for mediastinal schwannomas, providing both definitive diagnosis and curative treatment. Minimally invasive approaches, including video-assisted and robot-assisted thoracoscopic surgery, have increasingly replaced open thoracotomy because of reduced postoperative pain, shorter hospital stay, and excellent oncological outcomes (2, 4). In the present case, robotic-assisted thoracoscopic resection enabled complete excision of the lesion without complications.

## Conclusion

Mediastinal schwannomas may represent a diagnostic challenge when degenerative changes mimic necrotic lymphadenopathy on imaging studies. This case underscores the importance of integrated multimodal imaging and multidisciplinary evaluation in the assessment of mediastinal masses. Although contrast-enhanced CT, PET/CT and EBUS-TBNA provide valuable complementary information, definitive diagnosis often relies on surgical resection and histopathological confirmation.

## Patient perspective

The patient was informed throughout the diagnostic and therapeutic process and actively participated in the decision-making pathway. Although initially concerned about the possibility of malignancy, he expressed satisfaction with the multidisciplinary approach and the minimally invasive surgical treatment. Following surgery, the patient reported improvement of symptoms and was reassured by the benign nature of the lesion. At follow-up, he did not report procedure-related complications.

## Data Availability

The raw data supporting the conclusions of this article will be made available by the authors, without undue reservation.
